# Methods for Detecting Mycobacterial Mixed Strain Infections–A Systematic Review

**DOI:** 10.3389/fgene.2020.600692

**Published:** 2020-12-21

**Authors:** Alexander Stephen Byrne, Alex Goudreau, Nathalie Bissonnette, Isdore Chola Shamputa, Kapil Tahlan

**Affiliations:** ^1^Department of Biology, Memorial University of Newfoundland, St. John's, NL, Canada; ^2^Science & Health Sciences Librarian, University of New Brunswick, Saint John, NB, Canada; ^3^Sherbrooke Research and Development Center, Agriculture and Agri-Food Canada, Sherbrooke, QC, Canada; ^4^Department of Nursing & Health Sciences, University of New Brunswick, Saint John, NB, Canada

**Keywords:** *Mycobacterium*, tuberculosis, non-tuberculous, complex, mixed strain, infections, detection, methods

## Abstract

Mixed strain infection (MSI) refers to the concurrent infection of a susceptible host with multiple strains of a single pathogenic species. Known to occur in humans and animals, MSIs deserve special consideration when studying transmission dynamics, evolution, and treatment of mycobacterial diseases, notably tuberculosis in humans and paratuberculosis (or Johne's disease) in ruminants. Therefore, a systematic review was conducted to examine how MSIs are defined in the literature, how widespread the phenomenon is across the host species spectrum, and to document common methods used to detect such infections. Our search strategy identified 121 articles reporting MSIs in both humans and animals, the majority (78.5%) of which involved members of the *Mycobacterium tuberculosis* complex, while only a few (21.5%) examined non-tuberculous mycobacteria (NTM). In addition, MSIs exist across various host species, but most reports focused on humans due to the extensive amount of work done on tuberculosis. We reviewed the strain typing methods that allowed for MSI detection and found a few that were commonly employed but were associated with specific challenges. Our review notes the need for standardization, as some highly discriminatory methods are not adapted to distinguish between microevolution of one strain and concurrent infection with multiple strains. Further research is also warranted to examine the prevalence of NTM MSIs in both humans and animals. In addition, it is envisioned that the accurate identification and a better understanding of the distribution of MSIs in the future will lead to important information on the epidemiology and pathophysiology of mycobacterial diseases.

## Introduction

The genus *Mycobacterium* includes 192 different species with diverse growth characteristics and (Schulze-Röbbecke, 1993; Primm et al., 2004; Parte, 2018) and host tropism (Ahmed et al., 2013). Mycobacteria can be categorized into three major groups: those that cause tuberculosis (TB) and are part of the *Mycobacterium tuberculosis* complex (MTBC), those that cause leprosy (including *Mycobacterium leprae* and *Mycobacterium lepromatosis*), and the remaining members, commonly referred to as atypical mycobacteria (Siddiqi, 1978), non-tuberculous mycobacteria (NTM) or mycobacteria other than *M. tuberculosis* (MOTT) (Ahmed et al., 2013). In addition, members from this genus can be further categorized based on their growth rates into rapid and slow growers, where the latter have prolonged doubling times, making it challenging to cultivate them (Wayne and Kubica, 1986).

Tuberculosis is caused by *M. tuberculosis* infecting the lungs of the host, though the pathogen can also spread to other parts of the body (Sia and Wieland, 2011). Members of the MTBC such as *Mycobacterium africanum* also cause TB in humans (De Jong et al., 2010), while non-human host tropism is reported for other bacteria from the group. For example, *Mycobacterium bovis* causes bovine TB (Morris et al., 1994; Cosivi et al., 1998; Grange, 2001), *Mycobacterium caprae* can infect a variety of wild and domesticated animals and *Mycobacterium pinnipedii* causes TB in pinniped species (Roe et al., 2019). Tuberculosis is an ancient disease afflicting humans and *M. tuberculosis* has been studied for over a century, but the disease remains a significant cause of global morbidity and mortality (WHO, 2019). One reason why TB remains problematic is due to the complex interaction between MTBC members and their hosts, many aspects of which are still not fully understood. In addition, the emergence and spread of drug resistant forms of *M. tuberculosis* further exacerbates the situation, leaving few effective treatment options in some cases (WHO, 2019).

The NTM group comprises over 150 different species, including several pathogens from the *Mycobacterium avium* and *Mycobacterium abscessus* complexes (Tortoli, 2014). Members of the *M. avium* complex (MAC) are commonly found in the environment and cause opportunistic infections (Ichiyama et al., 1988; Von Reyn et al., 1993; Yajko et al., 1995; Reed et al., 2006), especially in immunocompromised individuals such as those suffering from acquired immunodeficiency syndrome (AIDS) (Jacobson et al., 1991; Havlik et al., 1992; Griffith et al., 2007). *M. avium* and *Mycobacterium intracellulare* (also a MAC member) cause pulmonary infections (Guthertz et al., 1989; Hocqueloux et al., 1998), where the latter can also infect immunocompetent individuals (Han et al., 2005; Koh et al., 2012). Furthermore, *M. avium* includes several subspecies (*avium, paratuberculosis, hominissuis* and *silvaticum*), which can infect organs other than the lungs (Thorel et al., 1990; Mijs et al., 2002). For example, *M. avium* subspecies *paratuberculosis* (MAP) causes Johne's disease in ruminants and has been linked to Crohn's disease in humans, conditions that afflict the small intestine (Behr and Kapur, 2008; Sweeney et al., 2012).

The *M. abscessus* complex includes three fast-growing subspecies (*abscessus, massiliense* and *bolletii*), which are highly resistant to many antibiotics and cause a wide range of human infections (Cho et al., 2013; Sassi and Drancourt, 2014; Lee et al., 2015; Adekambi et al., 2017). Another NTM of significance is *M. genavense*, an opportunistic pathogen that often causes disease in immunocompromised patients and has also been found to infect various domestic companion animals (Hoop et al., 1993; Böttger, 1994; Kiehn et al., 1996; Hughes et al., 1999; Krebs et al., 2000; Lucas et al., 2000; Hoefsloot et al., 2013). The NTM discussed above are just a few of many that are of concern to human and animal health (Piersimoni and Scarparo, 2008; Cook, 2010; Griffith, 2010; Atkins and Gottlieb, 2014; Biet and Boschiroli, 2014), demonstrating the propensity of members from this group to cause diverse diseases if given the opportunity.

**Figure 1:**
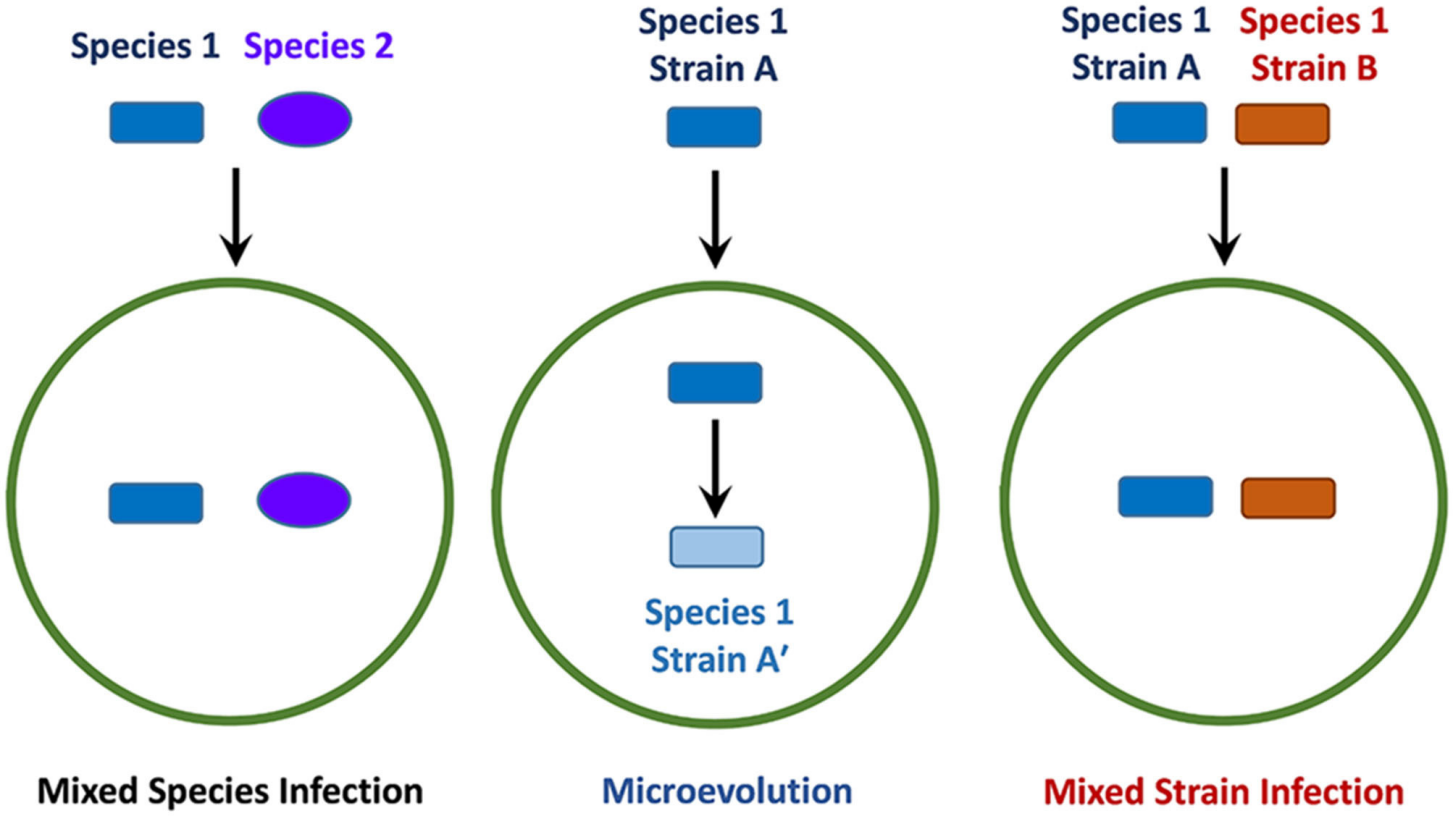
Schematic of different infection events involving pathogens from the same genus. The green circle represents a permissive host whereas the dark blue rectangle and purple oval indicate different species of pathogens belonging to the same genus. The rectangles of different colors indicate distinct strains derived from the same species, where blue indicates microevolution and red, mixed strain infection.

The progression and outcome of an infection is dependent on many factors, which include the resident host-microbiome and the presence of other pathogens, sometimes from the same genus (Figure 1) (Adami and Cervantes, 2015; Namasivayam et al., 2019, 2020). Mixed-species infections refer to the phenomenon where different species belonging to the same genus concurrently infect a single host (Figure 1). Another important factor to consider is the potential for genetically distinct strains (or isolates) of the same pathogenic species to infect a single host at any given time, which is sometimes referred to as an polyclonal infection (Taylor et al., 1997; Cohen et al., 2012; McNaughton et al., 2018). This situation can potentially arise if an isolate undergoes intra-host evolution (also referred to as microevolution) following infection (Figure 1), leading to minor genetic differences in the resulting progeny (Jordan et al., 2002; Feil, 2004; Ley et al., 2019). Another mechanism leading to polyclonal infections involves concomitant or sequential infection by genetically distinct strains (Figure 1), a process that is referred to as mixed strain infection (MSI). The presence of multiple strains with varying genotypes can result in altered physiological characteristics or pathogenicity, which in turn can affect transmission dynamics (Taylor et al., 1997), or lead to treatment complications due to varying antibiotic resistance profiles (also known as heteroresistance) (Van Rie et al., 2005; Shin et al., 2018). It is possible that a single treatment regimen will not be optimal against all strains in an individual, causing the infection to persist or return after briefly subsiding. MSIs are particularly relevant in slow-growing pathogens such as *M. tuberculosis*, MAC members and other related mycobacteria, as these organisms can remain undetectable for long periods of time (Whitlock and Buergelt, 1996; Gengenbacher and Kaufmann, 2012). If an MSI exists and the initial treatment is unsuccessful, the persistence of these infections may result in the development of more severe disease over time (Baldeviano-Vidalón et al., 2005). Additionally, MSIs have the potential to interfere with host immune responses due to antigenic variations that might exist between different strains (Huang et al., 2010; Cohen et al., 2012; Yoshida et al., 2018). Therefore, by examining MSIs and their transmission, successful treatment methods can be devised, and essential information might also be gained for use in future vaccine development endeavors.

The purpose of this study was to conduct a systematic review to gain a better understanding of MSIs across the genus *Mycobacterium* and the methods used to detect them. Typically, the detection of such infections is challenging due to the lack of distinct intraspecies markers that allow for discrimination between isolates/strains. Despite this, MSIs in mycobacteria were found using a variety of strain typing methods, each with a different level of discriminatory ability and ease of use, with different methods focusing on specific aspects of the *Mycobacterium* genome. Mycobacterial strain discrimination is made possible by the analysis of restriction fragment length polymorphisms (RFLP) in species specific insertion sequences (IS) such as IS*6110* (associated with the MTBC, with some exceptions) (Coros et al., 2008; Gonzalo-Asensio et al., 2018), and IS*1245* or IS*1311* (both associated with the MAC) (Guerrero et al., 1995; Johansen et al., 2005; Coll et al., 2014). Another general method used to discriminate between strains exploits the nucleotide sequences present in variable number tandem repeats (VNTR), which are dispersed throughout mycobacterial genomes. By examining differences in the number of nucleotide repeats present at distinct loci, individual strains can be typed. Different mycobacteria harbor a variety of VNTR loci, though depending on the species and loci examined, they may instead be referred to as multi-locus variable-number tandem repeats (MLV) (Overduin et al., 2004; Hill et al., 2012; Biffa et al., 2014), mycobacterial interspersed repetitive unit–variable number tandem repeats (MIRU-VNTR) (Supply et al., 2001, 2006) or short sequence repeats (SSRs) (Amonsin et al., 2004; Podder et al., 2015). Analysis of the entire genome at the individual nucleotide level using methods based on whole-genome sequencing (WGS) also allows for examination of strain diversity, but at a resolution unmatched by RFLP or VNTR based methods. By using WGS, strains can be typed and compared without focusing on a given set of loci allowing for more accurate detection of MSIs, re-infections, and relapses (Homolka et al., 2012; Coll et al., 2014; Witney et al., 2017; Lipworth et al., 2019). Heterogeneous (also referred to as heterozygous) single nucleotide polymorphisms (SNPs) are predominantly used in strain comparisons, and the presence of many different SNPs in isolates from a single sample is suggestive of MSIs (Sobkowiak et al., 2018).

It was also our intention to help clarify true MSIs as compared to similar events such as re-infection, relapses, and microevolution. While polyclonal infection may refer to microevolution, some studies have also used the term to describe infections that fit the criteria of an MSI (Adams et al., 2012; Fujita et al., 2014; Farmanfarmaei et al., 2017; Kamakoli et al., 2017, 2020b; Nathavitharana et al., 2017). Due to this lack of consensus regarding the terminology used in the literature and to be consistent in our study, we have selected definitions to describe the different events (Table 1). For our purposes, MSIs refer to an infection where multiple unrelated strains, which did not evolve from an initial infecting strain, are present within a single host at the same time.

**Table 1:** Glossary of terms.

**Term^a^**	**Description^b^**
Sample/specimen	A sputum, blood, feces or otherwise uncultured biological sample from a host/patient, which is directly used for strain-typing analysis or is subsequently used for culturing.
Culture	Refers to the amplification of bacteria following growth on solid or in liquid media using a sample/specimen as an inoculant. Does not explicitly imply a pure or axenic culture.
Isolate (strain, if characterized)	Refers to a single colony from an agar plate or an axenic bacterial culture derived from a sample/specimen.
Microevolution	A co-infection that involves multiple strains/sub-strains in a single host that evolved from a single strain that caused the initial infection.
Mixed genotype infection (MGI)/polyclonal infection	A co-infection caused by two or more strains of the same species in a single host. Encompasses both microevolution and mixed strain infections.
Mixed strain infection (MSI)	A co-infection caused by phylogenetically distinct strains of the same species in a single host (contrary to microevolution). Sometimes also referred to simply as mixed infection in the review.
Re-infection	Recurrent disease caused by a strain that is unrelated to the one that caused the initial infection.
Relapse	Recurrent disease due to the same strain that caused the initial infection.

a *Only non-standard terms or those that have been modified for use in the review are listed*.

b *Descriptions for the purposes as used in the current review*.

## Materials and Methods

**Figure 2:**
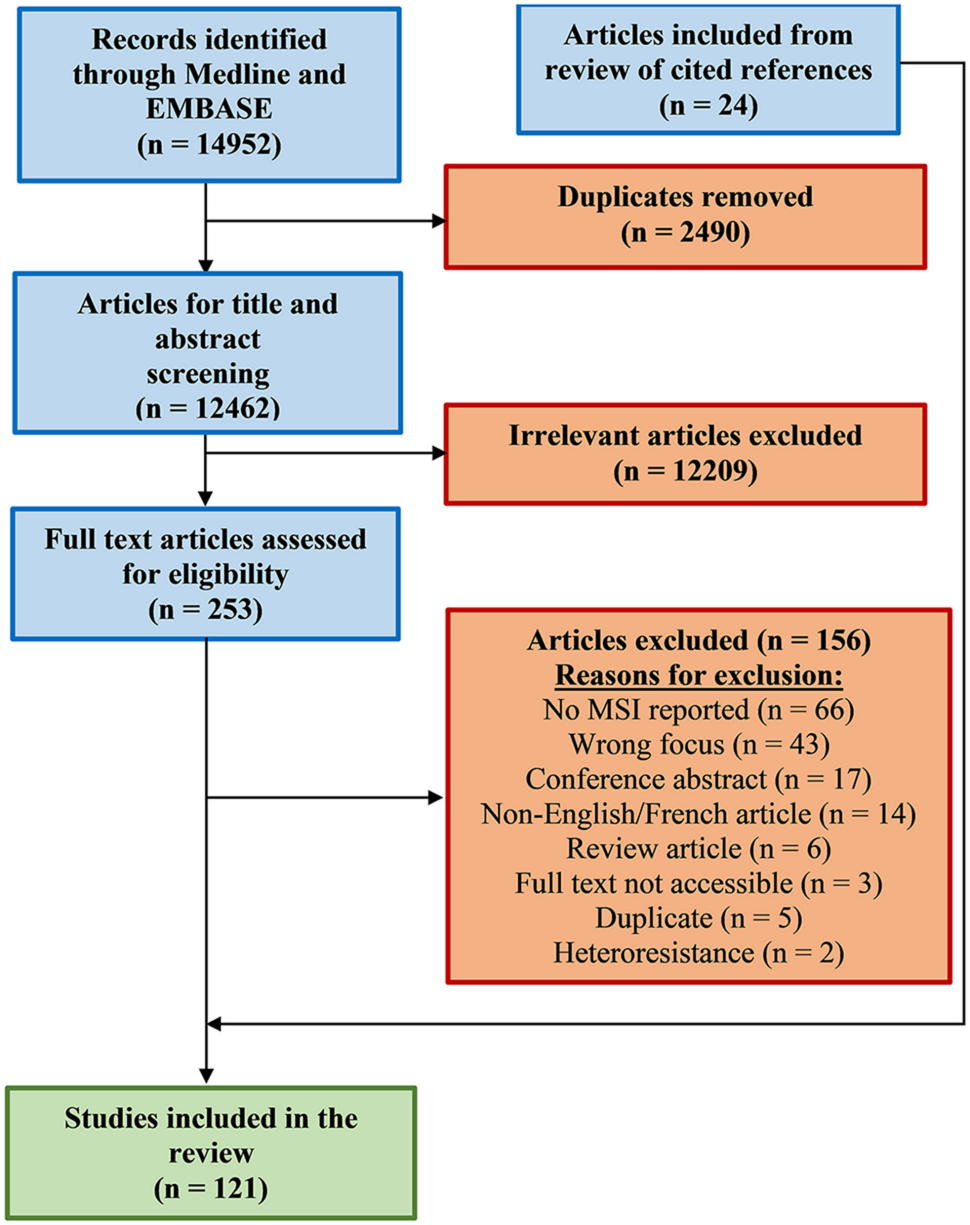
Flow diagram of search strategy. Modified preferred reporting items for systematic reviews and meta-analyses (PRISMA) results for search on mycobacterial MSI detection. Details are provided in material and methods section and Supplementary Table 1.

A systematic review of the literature was performed to identify methods used for detecting MSIs caused by a single species of mycobacteria. This process comprised four stages, where the first one entailed utilizing a comprehensive search strategy to locate published studies. An initial limited search was conducted in Ovid MEDLINE to compile a list of keywords and index terms from relevant articles. A full search strategy was then developed by a librarian by testing search terms in MEDLINE and only those terms yielding unique results were retained for further use. The full search strategy was externally peer-reviewed by a second librarian using the Peer Review of Electronic Search Strategies (PRESS) guidelines (McGowan et al., 2016). The search strategy was then adapted for EMBASE (Elsevier), following which both the MEDLINE and EMBASE were initially searched on June 11, 2020, with no prerequisite limits. An updated search was performed on August 25th, 2020 to include the term “polyclonal” (Supplementary Table 1). Search results were collated and uploaded into Endnote version X8.2 (Clarivate Analytics, PA, USA) for organization followed by Covidence™ (Veritas Health Innovation Ltd, Melbourne, Australia), a screening and extraction tool for systematic reviews. After removal of duplicates, reference lists of all selected studies were screened for additional articles of interest using Google Scholar (Figure 2). The second stage involved the screening of article titles and abstracts by two reviewers. The inclusion criteria for articles at this stage were as follows: (i) presence of both a title and abstract; (ii) abstracts in English or French; (iii) primary article or review; (iv) explicit mention of mixed infection or other synonymous terms like double infection, multiple infection, simultaneous infection or polyclonal infection; (v) described molecular or phenotypic methods; and (vi) mention of *M. tuberculosis* (MTBC), non-tuberculous mycobacteria (NTM), mycobacteria other than tuberculosis (MOTT), atypical mycobacteria, environmental mycobacteria or *M. leprae*. The presence of additional terms including “mixed, simultaneous, multiple, mycobacteria, concomitant, concurrent, co-infection, polyclonal, *Mycobacterium*, heterogeneity, subtype, sub-type, double, more than one, dual, and superinfection” were searched for within abstracts to assist in study identification. Articles were excluded if they were: (i) opinion pieces; (ii) commentaries; or (iii) not written in English or French. Conflicts on inclusion/exclusion of specific articles were resolved after analysis by a third reviewer or by mutual agreement by the team.

Full texts of articles were uploaded to Covidence™ and subsequently assessed for eligibility at the third screening stage using the following inclusion criteria: (i) full-text availability; (ii) article in English or French; and (iii) reported mixed infection, double infection, multiple infection, simultaneous infection or polyclonal infections involving one *Mycobacterium* species using molecular or phenotypic methods. Articles were excluded from the main list if: (i) they reported clonal variants suggestive of microevolution of a single strain; (ii) reported detecting mixed-species infections, e.g., *M. tuberculosis* and *M. bovis* together; (iii) article was unavailable; (iv) they were dissertations, conference or poster presentation abstracts; (v) did not contain sufficient information; or (v) were not in English or French. In the last stage of the analysis, initial findings from the full-text screening were analyzed to identify data for extraction and use in the review. All articles citing the identified reports for use in the review were also examined for relevant information, which was included in the final results.

## Results and Discussion

An initial screen of the literature yielded 14,952 records, and after the removal of duplicate and non-relevant entries based on abstracts and content, 253 articles were retained for full-text review (Figure 2). Examination of these articles resulted in the further exclusion of 156 entries for various reasons as described in Figure 2, leaving 97 reports for inclusion in the review. Additionally, 24 other relevant studies cited in the 97 reports were also included and are presented in the modified Preferred Reporting Items for Systematic Reviews and Meta-Analyses (PRISMA), to adhere to the systematic review format (Figure 2).

Data was extracted from all 121 selected articles, and general information, including the publication year, primary author, study location, bacterial and host species involved, was recorded (Supplementary Table 2). The number of samples/isolates, if they were derived from clinical specimens or cultures, the prevalence of MSIs reported in each study and the human immunodeficiency viruses (HIV) status of human subjects was also noted when possible. The studies were allocated into two separate groups based on MSI reports in humans and animals, respectively (Table 2). Reports were further classified based on the location of study by continent, publication year (grouped by decade), the analysis method used, and whether the study examined MSIs involving members of the MTBC or NTM. If a study examined multiple types of hosts or used different methods that successfully identified MSIs, then it was counted in more than one category. A brief overview of the different methods that successfully detected mycobacterial MSIs is presented in Table 3. It was noted that reports involving *M. tuberculosis* MSIs from humans were significantly overrepresented than those involving NTM or animals (Table 2). Thus, results from the analysis on MTBC and NTM are discussed separately to highlight methods and significant findings.

**Table 2:** General characteristics of reviewed mycobacterial studies involving MSIs (*n* = 121).

**Attribute**	**Human (%)^a^**	**Animal (%)^a^**
**Mycobacterial group responsible for MSIs**		
*M. tuberculosis* Complex (MTBC)	87 (71.9%)	8 (6.6%)
Non-tuberculous Mycobacteria (NTM)^b^	18 (14.9%)	9 (7.4%)
**Geographic location^c^**		
Africa^b^	28 (23.1%)	4 (3.3%)
Asia^b^	31 (25.6%)	0 (0.0%)
Europe^b^	20 (16.5%)	8 (6.6%)
North America	15 (12.4%)	3 (2.5%)
Oceania	1 (0.8%)	0 (0.0%)
South America^b^	11 (9.1%)	3 (2.5%)
**Publication year (decade)**		
Pre-2000	12 (9.9%)	0 (0.0%)
2000-2009^b^	32 (26.4%)	4 (3.3%)
2010-2019	54 (44.6%)	13 (10.7%)
2020	7 (5.8%)	1 (0.8%)
**MSI detection method^d^**		
Insertion Sequence Based RFLP^b^	34 (28.1%)	3 (2.5%)
Variable Number Tandem Repeat	44 (36.4%)	12 (9.9%)
Whole Genome Sequencing	12 (9.9%)	3 (2.5%)
Spoligotyping	14 (11.6%)	6 (5.0%)
Region Specific PCR^b^	16 (13.2%)	0 (0.0%)
Other^e^	11 (9.1%)	0 (0.0%)

a *Studies were grouped based on multiple criteria (host, geographical location, publication date and methods used). Totals for some sections may not add up to 100% as certain studies met multiple criteria and were therefore counted more than once*.

b *The subsection contains studies counted under more than one criterion*.

c *Excluding one study (Gan et al., 2016), which used information on isolates from a global database*.

d *Not counting methods that were unable to identify MSIs in the respective studies. Methods are briefly described in Table 3 and details are included in Supplementary Table 2*.

e *Includes methods such as phage typing, PFGE, etc. Details are provided in Supplementary Table 2*.

**Table 3:** Overview of methods that were used to detect mycobacterial MSIs.

**Method^a^**	**Target^b^**	**Output or readout^c^**	**Sample type^d^**
Insertion sequence based RFLP	Species specific insertion sequences	Differences in restriction enzyme band hybridization patterns	Culture
Variable number tandem repeat	Species specific tandem repeat loci	Differences in copy numbers based on PCR	Specimen or culture
Whole genome sequencing	Entire genome	Heterogeneous SNPs	Specimen or culture
Spoligotyping	Direct repeat spacers (CRISPR)	Differences in spacer sequences based on hybridization	Specimen or culture
Region-specific PCR	Chromosomal targets depending on the specific method and species	Differences in PCR band patterns	Specimen or culture
Phage typing^e^	Lysis of colonies by mycobacteriophages	Susceptibility to a select mycobacteriophages	Culture
Pulse field gel electrophoresis^e^	Enzymatic digestion of chromosomal DNA	Differences in restriction enzyme band patterns	Culture
pTBN12 digestion^e^	Species specific plasmid fingerprinting	Differences in restriction enzyme band patterns	Culture
*hsp65* PCR-RFLP^e^	*hsp65* gene	Differences in restriction enzyme band patterns	Culture
Random amplified polymorphic DNA^e^	Species specific chromosomal DNA	Differences in PCR band patterns	Culture
Other RFLP^e^	Species specific DNA sequences	Differences in restriction enzyme band hybridization patterns	Specimen or culture

a *References for methods are present in the main text and Supplementary Table 2*.

b *The component or property of the bacterial cell analyzed by the respective methods*.

c *The result obtained for interpretation using the respective methods*.

d *The type of material that can be used for the assay. Note that all methods except for phage typing use extracted DNA for analysis*.

e *Methods listed come under the “other” category in Table 2*.

### *M. tuberculosis* Complex

One of the earliest methods developed to discriminate between strains of *M. tuberculosis* involved the use of mycobacteriophages (Table 3), which specifically lyse certain strains, leading to the formation of plaques on solid agar plates (Jones and Greenberg, 1978). Phage typing has also been used to identify cross-contamination, transmission dynamics and MSIs based on the sensitivities of *M. tuberculosis* isolates to a panel of selective phages (Mankiewicz and Liivak, 1975; Bates et al., 1976; Snider et al., 1984; Jones, 1988), but more modern methods are faster and offer better discriminatory power (Snider et al., 1984). Our primary workflow did not find any reports on the identification of MSIs using phage typing, but secondary searches found two such studies. Mankiewicz and Liivak (1975) sampled 233 patients, of which 33 (14.2%) showed evidence of MSIs due to the presence of multiple *M. tuberculosis* phage types in a single culture. In the other study, Bates et al. (1976) analyzed samples from 87 different patients and identified three (3.4%) as having mixed phage-typing profiles. While the possibility that the presence of multiple *M. tuberculosis* phage types within the same patient could indicate an MSI, the limited discriminatory power of the method prevents definitive confirmation and cannot completely rule out intra-host microevolution.

Until recently, RFLP based on the insertion sequence IS*6110* was the standard method used for comparing the genetic relatedness of *M. tuberculosis* isolates (Van Soolingen et al., 1993, 1995). IS6*110* (sometimes referred to as IS*986*) belongs to the IS*3* family, members of which are only present in the MTBC (Hermans et al., 1990; Thierry et al., 1990a,b). *M. tuberculosis* and *M. bovis* strains can contain 0–25 and 0–3 copies each of IS*6110*, respectively (Cave et al., 1991, 1994; Van Soolingen et al., 1991; Yuen et al., 1993; Fomukong et al., 1994; Brosch et al., 2000; Lok et al., 2002; Singh et al., 2004; Steensels et al., 2013). Variations in the copy number of IS*6110* elements within different *M. tuberculosis* strains make it an attractive target for epidemio-typing isolates containing multiple copies of the insertion sequence, but not in low copy number strains. Therefore, IS6*110*-typing has led to the development of extensively used standardized protocols (Table 3) (Van Embden et al., 1993).

In total, 26 (21.5%) of the publications reported herein included the use of IS*6110*-typing methods for detecting MSIs involving MTBC members (Chaves et al., 1999; Pavlic et al., 1999; Yeh et al., 1999; Braden et al., 2001; Du Plessis et al., 2001; Richardson et al., 2002; García De Viedma et al., 2003, 2005; Allix et al., 2004; Das et al., 2004; Shamputa et al., 2004, 2006; Baldeviano-Vidalón et al., 2005; Cox et al., 2005, 2007; Van Rie et al., 2005; Van Der Zanden et al., 2007; Andrade et al., 2009; Mokrousov et al., 2009; Gardy et al., 2011; Navarro et al., 2011; Von Reyn et al., 2011; Adams et al., 2012; Cerezo et al., 2012; Huyen et al., 2012; De Almeida et al., 2019), 11 of which exclusively used IS*6110*-RFLP (Supplementary Table 2) and one study used IS*6110*-inverse polymerase chain reaction (PCR) (Mokrousov et al., 2009). All the studies employed cultures in their analysis, and IS*6110*-inverse PCR was primarily used as a tool to identify strains belonging to the Beijing evolutionary lineage (Mokrousov et al., 2003, 2009). The first report on MSI detection using IS*6110*-RFLP was by Yeh et al. (1999), who noticed multiple bands of varying intensities in a sample from a patient due to the presence of two separate strains. Another study found an MSI with two drug-susceptible isolates in an immunosuppressed patient (Pavlic et al., 1999), and similar infections with drug-susceptible and drug-resistant *M. tuberculosis* have also been detected by IS*6110*-RFLP in separate reports (Braden et al., 2001; Hofmann-Thiel et al., 2009). Overall, MSIs were identified in 0.4–100% of cases using IS*6110*-RFLP in these studies (Supplementary Table 2), where most reports with 100% MSI detection rates involved a single patient (Pavlic et al., 1999; Yeh et al., 1999; Andrade et al., 2009). In addition, a recent study detected MSIs in 3 out of 17 samples from patients using two probes for IS*6110*-RFLP instead of a single probe used conventionally (De Almeida et al., 2019).

Spacer oligotyping (spoligotyping) is a commonly used method for the simultaneous detection and identification of MTBC members (Table 3) (Kamerbeek et al., 1997). Spoligotyping exploits the nucleotide sequence diversity of clustered regularly interspaced palindromic repeats (CRISPRs) (Haft et al., 2005), which are present in many bacteria and archaea (Barrangou et al., 2007). The chromosomal locus specifically used in this assay is known as direct repeat (DR) in mycobacteria (Hermans et al., 1991). Spoligotyping is traditionally performed by amplifying the entire DR region using PCR with a pair of oligonucleotide primers, one of which is labeled with biotin to aid in the detection of products by hybridization. Membranes containing a unique set of 43 covalently bound synthetic oligonucleotide spacer sequences derived from *M. tuberculosis* and *M. bovis* Bacille Calmette-Guérin (BCG) are used in the hybridization (Kamerbeek et al., 1997), and can differentiate between MTBC isolates based on the presence or absence of spacers.

Spoligotyping has been widely used in epidemiological studies to investigate the cause of recurrent TB (defined as endogenous reactivation of an initially infecting strain or exogenous reinfection with a different strain) (Small et al., 1994; Warren et al., 2002; Van Rie et al., 2005; Andrews et al., 2008), tracking epidemics (Goyal et al., 1999; Källenius et al., 1999; Caminero et al., 2001), and investigating laboratory cross-contamination (Nivin et al., 2000). Twenty (16.5%) publications reported using spoligotyping as one of the methods for detecting MSIs, with a majority employing one (Pavlic et al., 1999; Cox et al., 2005; García De Viedma et al., 2005; Umubyeyi et al., 2007; Furphy et al., 2012; Biffa et al., 2014; Chaoui et al., 2014; Lamine-Khemiri et al., 2014; Ssengooba et al., 2015; Wang et al., 2015; Egbe et al., 2017; Baffoe-Bonnie et al., 2019) or two (García De Viedma et al., 2003; Mokrousov et al., 2009; Huyen et al., 2012; Navarro et al., 2015; Silva-Pereira et al., 2019) additional methods (Supplementary Table 2). Only three reports used spoligotyping as the sole genotyping method and reported MSIs at frequencies ranging from 11.8 to 57.1% (Andrews et al., 2008; Kamakoli et al., 2018; Guernier-Cambert et al., 2019).

One significant limitation of spoligotyping is that it can underestimate MSIs as hybridization signals from multiple strains in a sample can overlap and appear as a single pattern (Kamakoli et al., 2018). For this reason, when spoligotyping is used to investigate MSIs, subculturing is usually performed to obtain single isolated colonies for testing (García De Viedma et al., 2003; Shamputa et al., 2004; Huyen et al., 2012). The ability to detect MSIs in the latter case is dependent upon the proportion of different strains in the original sample and the number of colonies picked for analysis. To help resolve this problem, Lazzarini et al. (2012) developed a computational method that can predict if individual spoligotypes contained signatures from more than one of four major global lineages, which would indicate an MSI. In most cases, a secondary typing method like MIRU-VNTR, IS*6110*-RFLP or WGS may be required to verify results that may appear to contain a single spoligotyping pattern. It is worth noting that although spoligotyping can be applied directly to clinical specimens, all the studies reported herein used cultures, possibly due to the requirement of purified DNA for other methods employed by the authors (Shamputa et al., 2004, 2010; Hofmann-Thiel et al., 2009). Warren et al. (2004) used a combination of a lineage-specific PCR and spoligotyping to distinguish between *M. tuberculosis* strains belonging to the Beijing- and non-Beijing evolutionary lineages. They detected MSIs in 19% of samples from patients associated with retreatment in their study. In addition, WGS was used as second technique in three spoligotyping based studies (Witney et al., 2017; Baffoe-Bonnie et al., 2019; Silva-Pereira et al., 2019), one of which focused on animals. In their work, Silva-Pereira et al. (2019) detected an *M. pinnipedii* MSI in a South American sea lion (*Otaria flavescens*) using WGS, which was not suggested by *in silico* spoligotyping or MIRU-VNTR initially. The above-mentioned studies demonstrate the importance of using more discriminatory methods along with traditional screening techniques to ensure the detection of different strains that might be present in a single sample.

Variable number tandem repeats (VNTR) are short DNA sequences (Nakamura et al., 1987), which are dispersed throughout the genomes of many bacteria and eukaryotes (Cox and Mirkin, 1997). They vary in repeat unit length and repeat number depending on the specific organism and locus being analyzed (Supply et al., 1997). Since the repeat unit length at specific VNTR loci is known for each species, determining the numbers of repeats present at the respective loci can be used to discriminate between strains (Table 3). The use of VNTRs for typing *M. tuberculosis* strains was first reported in 1998 (Frothingham and Meeker-O'Connell, 1998), and since then the discrimination power has been improved by using combinations of mycobacteria-interspersed repetitive units (MIRUs) located at different loci throughout the genome (Smittipat and Palittapongarnpim, 2000; Le Flèche et al., 2002; Roring et al., 2002; Skuce et al., 2002). Initially, 12-locus MIRU-VNTR was used widely (Supply et al., 2001), but the method had some limitations in its ability to discriminate between unrelated isolates (Supply et al., 2006). To overcome this problem, the stability and resolution power of 29 MIRU-VNTR loci was evaluated using predominant global *M. tuberculosis* lineages, resulting in the standardization of 24-MIRU-VNTR loci for high-resolution epidemiological studies (Supply et al., 2006). In addition, the 15 most discriminatory loci of the 24 were selected for use in routine epidemiological investigations involving *M. tuberculosis* (Supply et al., 2006).

To generate a MIRU-VNTR profile, several genomic regions known to contain VNTRs are amplified by PCR using specific primer pairs either individually (simplex) or in multiples (multiplex). In this way, the number of repeats at each VNTR locus can be determined using different DNA sizing techniques for comparing isolates. It was found that 50 studies used VNTR-typing to detect *M. tuberculosis* MSIs, where 28 studies used it as the sole discriminatory method for this purpose (Allix et al., 2004; Shamputa et al., 2004; Umubyeyi et al., 2007; Fang et al., 2008; Hofmann-Thiel et al., 2009; Mokrousov et al., 2009; Stavrum et al., 2009; Dickman et al., 2010; Mulenga et al., 2010; Cohen et al., 2011; Gardy et al., 2011; Navarro et al., 2011, 2015; Cerezo et al., 2012; Furphy et al., 2012; Huyen et al., 2012; Hingley-Wilson et al., 2013; Muwonge et al., 2013; Peng et al., 2013; Biffa et al., 2014; Chaoui et al., 2014; Lamine-Khemiri et al., 2014; Zetola et al., 2014; Barletta et al., 2015; Mei et al., 2015; Pang et al., 2015; Shin et al., 2015, 2018; Ssengooba et al., 2015; Streit et al., 2015; Wang et al., 2015; Zheng et al., 2015; Antusheva et al., 2016; Hajimiri et al., 2016; Hu et al., 2016; Egbe et al., 2017; Farmanfarmaei et al., 2017; Ghielmetti et al., 2017; Kamakoli et al., 2017, 2019, 2020a,b; Khosravi et al., 2017; Kontsevaya et al., 2017; Nathavitharana et al., 2017; Sadegh et al., 2017; Silva-Pereira et al., 2019; Abascal et al., 2020; Baik et al., 2020; Sichewo et al., 2020). In addition, 24-locus MIRU-VNTR was the most commonly used method for detecting *M. tuberculosis* MSIs, and nearly all (47/50, 94.0%) of the reports used some form of culturing for analysis. Comparatively, MIRU-VNTR detected more MSIs than any other method based in the current review (Table 2). One reason for this could be the use of PCR amplification during MIRU-VNTR, which increases the sensitivity and detection power of the method (Shamputa et al., 2006), especially in instances where different strains are not proportionally present in a single sample.

Among other PCR-based methods used to detect MSIs, the majority were focused on differentiating between *M. tuberculosis* strains belonging to the Beijing and non-Beijing evolutionary lineages (Warren et al., 2004; Van Rie et al., 2005; Huang et al., 2010; Wang et al., 2011; Mustafa et al., 2016). Of the 121 studies we examined, 13 (10.7%) used region-specific PCR methods and detected MSIs in 3.2–100% of samples (Supplementary Table 2) (Theisen et al., 1995; Lourenço et al., 2000; García De Viedma et al., 2003; Warren et al., 2004; Baldeviano-Vidalón et al., 2005; Van Rie et al., 2005; Huang et al., 2010; Mallard et al., 2010; Wang et al., 2011; Hanekom et al., 2013; Peng et al., 2013; Müller et al., 2014; Mustafa et al., 2016). For example, Warren et al. (2004) detected MSIs in 35 (18.8%) of the 186 sputum cultures from TB patients tested during an epidemiologic study in South Africa. They reported that MSIs were more often associated with retreatment (23%) *vs*. new cases (17%), and the sensitivity and specificity of their method was comparable to IS*6110*-RFLP and spoligotyping (Warren et al., 2004). Using the same method, Van Rie et al. (2005) also detected one case of MSI with drug-susceptible and drug-resistant *M. tuberculosis* isolates. While the method developed by Warren et al. (2004) used simplex PCR, Huang et al. (2010) utilized multiplex PCR to detect MSIs caused by Beijing and non-Beijing lineage strains in 11.3% of the 185 sputum samples from patients without any prior history of TB treatment. Another group used quantitative PCR to detect MSIs based on the presence of both Beijing and non-Beijing lineages in 3% of TB patients <25 years in age using clinical specimens and cultures (Wang et al., 2011), whereas *M. tuberculosis* isolates belonging to the two lineages were also detected together in 14.7% cases by Mustafa et al. (2016). In contrast, a Latin American and Mediterranean (LAM) and non-LAM lineage-based PCR found MSIs in 4 out of 160 (2.5%) culture-positive sputa analyzed (Mallard et al., 2010), suggesting that such methods are useful in identifying MSIs under settings where the occurrence of *M. tuberculosis* isolates from mixed lineages is high. Therefore, by using an in-house PCR to identify isolates from the Beijing, Haarlem, S-family, and LAM evolutionary lineages, Hanekom et al. (2013) were able to detect MSIs in 31 (15%) of the 206 samples analyzed in their study.

*M. tuberculosis* MSIs have also been reported in patients with discordant drug susceptibility profiles on more than one occasion. Isolates from 10 such TB patients out of 89 (11.2%) were confirmed to have MSIs using Beijing lineage-specific PCR (Chen et al., 2007) and 16-locus MIRU-VNTR (Peng et al., 2013). Other techniques such as linker PCR (Theisen et al., 1995) and *gyr*A PCR/sequencing (Sreevatsan et al., 1997; Müller et al., 2014) have also detected *M. tuberculosis* MSIs in cases involving discordant drug susceptibility profiles and also in archaeological samples. In addition, two studies employed double repetitive element PCR (Lourenço et al., 2000; Baldeviano-Vidalón et al., 2005) based on the IS*6110* and a GC-rich repetitive sequence described by Friedman et al. (1995). Baldeviano-Vidalón et al. (2005) also observed multiple discrepancies in drug susceptibility testing results among follow up samples from patients, which were attributed to MSIs based on IS*6110*-RFLP analysis. Such studies emphasize the importance of considering MSIs during TB drug susceptibility testing and while devising appropriate treatment regimens.

Methods based on WGS provide the ability to examine strain diversity at very high resolution, which cannot be achieved by other techniques such as RFLP or MIRU-VNTR. Heterogeneous SNPs are predominantly used for strain discrimination, and the presence of many different SNPs in isolates from a single sample is suggestive of MSIs (Sobkowiak et al., 2018). While the concept is simple–a SNP is confirmed within several sequencing reads used to assemble the locus being examined (Table 3), the technical criteria for identifying bona fide SNPs varies between studies. Factors that can affect SNP detection include the quality and depth of sequencing, experimental design, sample preparation and pathogen species, to name a few (Hatherell et al., 2016). Some reports require that the frequency of the alternative base at a specific locus be found from anywhere between 5 and 30% or more of the reads for SNP calling (Bryant et al., 2013a; Guerra-Assuncaõ et al., 2015; Dippenaar et al., 2019), or even just two reads in some deep sequencing studies (Gan et al., 2016). Some studies also include threshold nucleotide base quality scores to minimize artifacts (Bryant et al., 2013a; Gan et al., 2016; Nimmo et al., 2020). Additionally, several loci with heterogeneous bases must be identified between isolates to qualify them as MSIs. However, the minimum number of SNPs used to qualify the presence of an MSI using isolates also varies, as anywhere over 16 to 80 have been used for the purpose depending on the sequencing technology (Bryant et al., 2013a; Gan et al., 2016). In addition, WGS analysis cannot be performed directly on clinical samples in most cases because the genomic complexity of the sample limits the confidence at which SNPs are called. Therefore, WGS often requires axenic cultures for strain typing and MSI identification (Van Den Berg et al., 2005; Döpfer et al., 2008; Davidson et al., 2016).

Of the 97 studies identified in Covidence, eight (8.2%) primarily used WGS to detect MSIs in humans (Bryant et al., 2013a; Gan et al., 2016; Witney et al., 2017; Baffoe-Bonnie et al., 2019; Dippenaar et al., 2019; O'Donnell et al., 2019; Anyansi et al., 2020; Wollenberg et al., 2020), while three others (3.1%) used the method to detect MSIs in various animal species (Davidson et al., 2016; Pfeiffer et al., 2017; Silva-Pereira et al., 2019), only one of which focused on a MTBC bacteria (Silva-Pereira et al., 2019). Secondary searches found four more human studies on WGS and mycobacterial MSIs (Chan et al., 2013; Kay et al., 2015; Dheda et al., 2017; Nimmo et al., 2020), in addition to reports where the method was used to confirm such infection that were initially identified using other means (Supplementary Table 2). For example, six isolates from 47 paired patient samples taken before and after treatment during the REMoxTB clinical trial were initially classified as relapses or re-infections by MIRU-VNTR but were later determined to be MSIs by WGS analysis (Bryant et al., 2013a). A follow-up study re-examined the same data using QuantTB (Anyansi et al., 2020), a tool developed to identify MSIs through the iterative comparison of SNPs and suggested that only four of the six MSI cases could be classified as such. O'Donnell et al. (2019) used isolates from a patient where drug susceptibility testing alluded toward an MSI involving susceptible and resistant *M. tuberculosis*, which was confirmed using WGS. They showed that the patient was initially infected with a drug-susceptible strain followed by an extensively drug-resistant *M. tuberculosis* isolate, which was selected for during antibiotic therapy. The high resolution of WGS underscores its importance in strain typing for devising individualized treatments for TB infections, although its widespread use may be limited in many high TB burden settings due to insufficient resources or technical capabilities. The ability to use traditional culture based drug susceptibility testing methods have limitations for many slow growing pathogenic mycobacteria, but genomics based technologies are more rapid and allow for the detection of resistance based on the presence of conferring mutations (Nicol and Cox, 2019). While PCR methods targeting specific genes can detect important drug resistance mutations for early diagnosis, WGS can additionally infer potential resistance, allowing for individualized treatment regimens (Nicol and Cox, 2019).

The use of WGS also enhances the detection of potential MSIs. For example, while examining pre-and post-treatment isolates from a TB patient, Witney et al. (2017) noticed 57 SNP differences between two of them, indicating a re-infection by a second strain. More detailed analysis of the WGS data indicated a potential MSI in the pre-treatment sample at a 3:1 genotypic ratio by two strains, where the minor genotype was closely related to the post-treatment isolate. This suggested that recurrent disease was caused by a relapse, where one of the two strains from the MSI was eliminated during the initial course of therapy. Interestingly, 24-locus MIRU-VNTR typing did not detect the genotype of the post-treatment isolate in the pre-treatment sample, which is intriguing, as the method was previously shown to detect MSIs in proportions as low as 1:99 (García De Viedma et al., 2005). Such reports suggest that many MSIs might have gone undetected due to technical limitations and could have potentially affected disease outcomes.

Although more TB MSIs are now being reported, evidence from archaeological studies indicate that the phenomenon has been around for a long time. Through metagenomics analysis of ancient DNA, one study identified an MSI (difference of 398 SNPs) within an eighteenth-century Hungarian mummy (Chan et al., 2013). A follow up study by a related group reported five MSIs in eight mummified bodies examined from the same archaeological site using similar metagenomics-based methods (Kay et al., 2015). In addition, a separate study employed *gyr*A PCR to successfully identify an MSI using ancient DNA from a variety of archaeological samples across Britain and France (Müller et al., 2014). The detection of MSIs using ancient DNA and complex samples is intriguing. Such reports also provide precedence for using metagenomics and other technologies to examine the prevalence and impact of such infections in future prospective and retrospective studies.

### Non-tuberculous and Other Mycobacteria

Our review showed that MSIs involving NTM have not been investigated to the same extent as compared to those caused by *M. tuberculosis* (Table 2). Amongst the 121 mycobacterial studies identified, 26 (21.5%) examined NTM, of which 17 (14.0%) and eight (6.6%) found MSIs in humans and animals, respectively, whereas one report identified MSIs in both human (Arbeit et al., 1993; Slutsky et al., 1994; Von Reyn et al., 1995; Devallois and Rastogi, 1997; Picardeau et al., 1997; Wallace et al., 1998; Legrand et al., 2000a,b; Oliveira et al., 2000; Saad et al., 2000; Dvorska et al., 2002; Panunto et al., 2003; Ohkusu et al., 2004; De Sequeira et al., 2005; Fujita et al., 2014; García-Pedrazuela et al., 2015; Kimizuka et al., 2019) and animal (Dvorska et al., 2007; Shitaye et al., 2008; Furphy et al., 2012; Gerritsmann et al., 2014; Johansen et al., 2014; Gioffré et al., 2015; Podder et al., 2015; Davidson et al., 2016; Pfeiffer et al., 2017) populations simultaneously (Pate et al., 2008). Many early studies used pulsed field gel electrophoresis (PFGE) to identify MSIs in 14.3–100% of patients infected with MAC bacteria (Arbeit et al., 1993; Slutsky et al., 1994; Von Reyn et al., 1995; Wallace et al., 1998; Ohkusu et al., 2004). PFGE was also used as the sole method to identify MSIs associated with other NTMs. For example, Legrand et al. (2000a) found 33.3% *M. simiae* MSI prevalence (3 of 9 hosts) in their study involving AIDS patients. In addition, PFGE was used in combination with other methods in studies on NTM MSIs. A report by Picardeau et al. (1997), detected 3 *M. avium* MSIs while examining 93 samples from AIDS patients using simple double repetitive element PCR (*Ma*DRE-PCR), which amplifies a region of the *M. avium* chromosome between IS*1245* and IS*1311* (Picardeau and Véronique, 1996). They confirmed these results using IS*1245*-RFLP, which showed the presence of multiple low-intensity bands in the same sample. Pulsed field gel electrophoresis was also able to pick up multiple banding patterns, including in some samples from other patients (Picardeau et al., 1997), but the criteria for attributing them to different strains (≥3 differences) was not surpassed (Tenover et al., 1995). A similar study on 31 AIDS patients from the Caribbean islands initially identified three potential polyclonal *M. avium* infections based on IS*1245*-RFLP, but PFGE analysis showed that two isolates had identical banding patterns (Legrand et al., 2000b). Therefore, results from PFGE analysis do not corroborate those obtained by other methods on multiple occasions, which might allude to differences in their discriminatory powers.

IS*1245* and IS*1311* have also been used as general fingerprinting targets to identify an MSI (25%) involving MAC members from 25 patients (Devallois and Rastogi, 1997). In a separate study, Oliveira et al. (2000) used RFLP analysis of *hsp*65 PCR products, IS*1245* and IS*1311*, respectively, to detect an *M. avium* MSI. Though the presence of IS*1245*-RFLP may be supplemented using additional methods, studies have shown it alone is capable of identifying 2.6–100% of NTM MSIs in samples from human subjects, some with HIV-AIDS (Supplementary Table 2) (Picardeau et al., 1997; Saad et al., 2000; Dvorska et al., 2002; Panunto et al., 2003; Pate et al., 2008). In addition, a study examined 41 samples from 14 AIDS patients using *Ma*DRE-PCR, which found four cases with multiple banding patterns, though re-evaluation using IS*1245*-RFLP only confirmed two as *M. avium* MSIs (De Sequeira et al., 2005). Based on the reports mentioned above, the feasibility of using IS*1245*-RFLP as a screening method for detecting NTM MSIs warrants further evaluation.

Kimizuka et al. (2019) have also used a variety of VNTR loci, including 16 *M. avium* tandem repeats (MATR) and 5 Higashi Nagoya tandem repeats (HNTR) to identify *M. avium* MSIs in samples from nine patients (13.8%) out of 65 examined. Another study examined samples from 120 patients with pulmonary MAC infections (94 *M. avium* and 26 *M. intracellulare*) (Fujita et al., 2014). MIRU-VNTR analysis using 15 loci for *M. avium* and 16 loci for *M. intracellulare* successfully identified 20 and 7 cases of *M. avium* and *M. intracellulare* MSIs, respectively. Other methods that have detected NTM MSIs in humans include random amplified polymorphic DNA (RAPD) analysis (Zhang et al., 1997). García-Pedrazuela et al. (2015) used the method and found MSIs involving several species including *M. abscessus, Mycobacterium chelonae, Mycobacterium fortuitum* and *Mycobacterium mucogenicum* while examining 64 isolates from Spanish patients.

Systematic screening using the Covidence platform also identified some reports on NTM MSIs in animals, including a few involving *M. avium* subsp. *paratuberculosis* (MAP). Gerritsmann et al. (2014) examined 39 MAP containing samples from a variety wild and domestic ruminant species, five of which were classified as MSIs using 8-locus MIRU-VNTR by them (Supplementary Table 2). In another study, Gioffré et al. (2015) used 8-locus MIRU-VNTR to identify a MAP MSI from a group to 97 cattle, sheep and goats based on differences in two loci. MAP MSIs have also been identified using multi-locus short sequence repeat (MLSSR or SSR) typing (Podder et al., 2015), which examines sequences that vary between isolates and allows for their discrimination (Amonsin et al., 2004). In addition, the use of DNA detection and sizing techniques such as fragment analysis of labeled PCR products further improves the resolution or SSR typing (Oakey et al., 2014; Podder et al., 2015). Using this strategy, Podder et al. (2015) identified MAP MSIs in all 18 animals from their study, which was subsequently confirmed by WGS analysis using some of their isolates (Davidson et al., 2016). MAP strains from the same animal had significantly different SNPs at high frequencies, which ruled out microevolution based evolutionary rates (Bryant et al., 2016).

In addition to MAP, there were reports on MSIs due to other NTM. *M. avium* subsp. *avium* MSIs were identified in domestic chickens using IS*901*-RFLP, where multiple banding patterns were detected in 7 of the 16 (43.8%) tissue samples tested (Shitaye et al., 2008). Eight-locus VNTR was used to detect *M. avium* subsp. *hominissuis* MSIs in pigs using isolates from multiple organs of a single animal (Johansen et al., 2014). Another study that previously reported a single *M. avium* subsp. *hominissuis* MSI in a human (out of 26 patients, 3.8%) using IS*1245*-RFLP also detected similar infections in 33 pigs (13.5%) (Pate et al., 2008). WGS was also used to detect non-MAP MSIs from 113 birds by Pfeiffer et al. (2017). They reported 12 cases of MSIs (2 involving *M. avium* and 10 involving *M. genavense*) based on differences in at least 12 SNPs as suggested by Walker et al. (2013). Therefore, NTM MSIs seem to occur in a variety of animal species, but studies examining their prevalence and impact are few and far between.

Our review did not reveal the occurrence of MSIs within leprosy causing mycobacteria. However, using artificially co-infected armadillos, Shin et al. (2015) demonstrated that distinct strains of *M. leprae* could simultaneously exist in the same host. They noted that the *in vivo* growth rate of the non-armadillo strain was significantly higher in the absence of competing strains, suggesting that pathological variations exist between the different strain types. Due to the challenges associated with culturing *M. leprae* (Scollard et al., 2006), the same experimental approaches used to study MTBC and NTM members might not be feasible, thereby limiting the potential for detecting *M. leprae* MSIs. Therefore, with the advent of more sensitive and powerful discriminatory methods, it is possible that the use of culture-independent techniques such as metagenomics will help to shed light on MSIs involving *M. leprae* in the future.

### Challenges Faced in Defining MSIs

Many questions are raised regarding the use of standard strain typing methods for delineating true MSIs from microevolution. When using IS*6110*-RFLP and MIRU-VNTR on *M. tuberculosis* isolates derived from the same host, MSIs are identified based on differences in specific DNA/PCR fragment profiles (Table 4). IS*6110*-RFLP profiles that differ by at least 2–3 bands indicate the occurrence of concomitant infection by distinct strains (Shamputa et al., 2004, 2006; Marx et al., 2014). Conversely, minor variations, i.e., anything <2 discriminatory bands, is considered as microevolution. In the case of MIRU-VNTR, MSIs are predicted based on heterogeneity at two or more loci (Shamputa et al., 2004, 2006; Cohen et al., 2011; Maghradze et al., 2019), whereas differences in a single locus suggests microevolution (Shamputa et al., 2004, 2006; Maghradze et al., 2019). Therefore, allelic diversity at more than one locus is a criterion for differentiating microevolution from true MSI when using MIRU-VNTR.

**Table 4:** Thresholds for distinguishing MSIs from microevolution events as per reviewed studies.

**Year^a^**	**Method^b^**	**Microevolution^c^**	**MSI^c^**	**References**
2004	IS*6110* RFLP	1-3 different bands	>3 different bands	Shamputa et al., 2004
	MIRU-VNTR	Double allele at 1 locus	Double alleles at ≥2 Loci	
2011	MIRU-VNTR	Double allele at 1 locus	Double alleles at ≥2 Loci	Cohen et al., 2011
2013	WGS	<80 heterogeneous SNPs	>80 heterogeneous SNPs	Bryant et al., 2013b
2014	IS*6110* RFLP	1-2 different bands	>2 different bands	Marx et al., 2014
2015	WGS	≤140 heterogeneous SNPs	>140 heterogeneous SNPs	Guerra-Assuncaõ et al., 2015
2019	MIRU-VNTR	Double allele at 1 locus	Double alleles at ≥2 Loci	Maghradze et al., 2019
2019	WGS	0-5 heterogeneous SNPs	757-833 heterogeneous SNPs	Dippenaar et al., 2019

a *Year of publication in chronological order*.

b *Details regarding specific methods are included in the main text and Supplementary Table 2*.

c *Thresholds or criteria used by the respective methods to differentiate between the two forms of infection*.

Defining MSIs using WGS based methods also has inherent challenges. However, the high discriminatory power of WGS allows for more definitive explanations for TB recurrence post-treatment (Bryant et al., 2013a; Guerra-Assuncaõ et al., 2015; Dippenaar et al., 2019). The classification of infections based on SNP differences between isolates from a single patient varies, where 5–10 suggest the occurrence of a relapse and anything over 100, a re-infection (Bryant et al., 2013a; Guerra-Assuncaõ et al., 2015; Dippenaar et al., 2019) (Table 4). These differences in SNP numbers are apparently set by taking into account the calculated evolutionary rate of *M. tuberculosis*, which ranges from 0.3 to 0.5 SNPs per genome per year, and limits the number of SNPs that can accumulate within strains in a defined period of time (Bryant et al., 2013b; Ford et al., 2013; Roetzer et al., 2013; Walker et al., 2013). Varying SNP thresholds have been used to define MSIs, where Bryant et al. (2013a) called them as such if isolates derived from the same patient had 80 or more different SNPs based on manual inspection. In comparison, Guerra-Assuncaõ et al. (2015) used a threshold of 140 heterogeneous SNPs to define MSIs based on an empirical cutoff generated during their data analysis. Furthermore, Dippenaar et al. (2019) did not define a specific threshold, as the numbers of heterogeneous SNPs in their study were either limited (1–2, classified as microevolution), or rather numerous (757–883, classified as MSI). These differences in SNP numbers used to identify *M. tuberculosis* MSIs shows that the field requires some form of standardization so that results can be compared between studies. It is possible that the lack of a clear definition for MSIs using WGS could hinder the identification of some reports for inclusion in this review. Although this is unlikely as only a few studies have used WGS technology for such purposes to date, and most of them were manually screened for their relevance.

Considering that the criteria necessary for identifying different types of *M. tuberculosis* polyclonal infections using IS*6110*-RFLP and MIRU-VNTR are well-defined, several studies were also found in the current review that reported on microevolution (Martín et al., 2007; Streit et al., 2015; Cohen et al., 2016; Jajou et al., 2018). The classification was not evident in some other reports that used IS*6110*-RFLP or MIRU-VNTR, due to results potentially indicating either an MSI or a microevolution event depending on the applied criteria (Al-Hajoj et al., 2010; Alves et al., 2011), or due to WGS not having a well-defined SNP threshold (Liu et al., 2015). While microevolution may initially appear to have little significance when compared to MSIs in terms of virulence and pathogenicity, one study found that the presence of highly evolvable repeats near genes (VNTR52, QUB26, or MIRU10/27) can influence gene expression in different *M. tuberculosis* isolates (Pérez-Lago et al., 2013). In addition, microevolution has obvious implication on the emergence of drug resistance due to selective pressures applied during treatment regimens (Fonseca et al., 2015).

Microevolution derived infections may not be as distinct as MSIs, but different clonal variants can still spread in unique patterns. While a strain undergoes a genetic drift, some of the progeny might spread both within and between hosts. For example, WGS identified separate *M. tuberculosis* clonal variants derived from within host microevolution at both respiratory and extrapulmonary sites in a patient (Pérez-Lago et al., 2014; Ssengooba et al., 2016). A study by Buff et al. (2009) examined several TB cases where IS*6110*-RFLP and spoligotyping of isolates from community transmission events showed identical patterns but exhibited variations at a single locus during 12-locus MIRU-VNTR analysis. It was shown that *M. tuberculosis* isolates from the source patient also displayed varying MIRU-VNTR profiles that matched the secondary patients, suggesting that different clonal variants were transmitted individually.

In addition to the MSIs described in our review, there is also evidence for the microevolution of mycobacterial pathogens in animals. For example, microevolution events and MSIs involving *M. avium* subsp. *hominissuis* in bongo antelopes were identified using 8-locus MIRU-VNTR and IS*1245*-RFLP (Moravkova et al., 2013). Another study used 8-locus MIRU-VNTR to examine *M. caprae* from 55 different animal hosts (including goats, cattle, sheep and wild boar) in Portugal and identified microevolution derived infections in 12 of them (Reis et al., 2020). Therefore, mycobacterial microevolution also occurs in animals, which is expected given the current state of knowledge regarding such infections in humans.

## Conclusion

As summarized in this systematic review, a variety of terminologies have been used for describing MSIs, some of which overlap with microevolution. Findings show that MSIs exist among many different mycobacteria, although the majority of studies have been conducted in humans and predominantly focus on *M. tuberculosis*. In addition, most studies used VNTR based methods, though more recent reports involved WGS. This change in methodology might represent an overall shift as newer technologies are developed and used more widely for strain typing. With methods suitable for large-scale screening of genetic variations by the massively-parallel sequencing approaches, the potential for the accurate identification of MSIs is now accessible at lower cost. Methods based on WGS offer unprecedented resolution, but appear to lack uniform SNP thresholds (Bryant et al., 2013a; Guerra-Assuncaõ et al., 2015; Dippenaar et al., 2019), sometimes making it ambiguous to differentiate between a true MSI or microevolution event. Therefore, there is scope for further standardizing WGS criteria for discriminating MSIs from genomic drift or technical aberrations, but as of now a universal definition for calling mycobacterial MSIs using the technology is lacking. Consideration of both microevolution and MSI has potential implications for developing personalized medical treatments for diseases such as TB. A select number of publications examined the historical context of *M. tuberculosis* using ancient DNA (Chan et al., 2013; Müller et al., 2014; Kay et al., 2015), suggesting that MSIs have likely been around for a long time. Furthermore, reports on discrepant drug susceptibilities between one or more *M. tuberculosis* isolates from the same host during a single disease episode underscores the importance of considering MSIs when managing mycobacterial infections. Reports have shown that TB patients with MSIs associated with heteroresistance are at a higher risk of poor treatment outcomes (Zetola et al., 2014; Cohen et al., 2016; Kamakoli et al., 2017; Shin et al., 2018). Therefore, additional strain typing is recommended under certain incidences where heteroresistance is detected, to determine if it is caused due to MSIs or microevolution. It was also noted that studies on NTM MSIs in humans and animals are limited, but such infections are found across many species. Given the importance of NTM in causing opportunistic and nosocomial infections in humans, and diseases in farmed animal, the prevalence and impact of MSIs caused by this large and important group of mycobacteria warrants further investigation.

## Data Availability Statement

The original contributions presented in the study are included in the article/Supplementary Material, further inquiries can be directed to the corresponding authors.

## Author Contributions

IS, AB, and KT developed the framework for the review. AG formulated the search strategy used to identify publications, with some input on key terms from AB, IS, and KT. AB and IS performed primary screening in Covidence, with KT resolving any conflicts on paper inclusion. Data extraction and analysis was conducted by AB and IS. AB, IS, and KT performed writing and primary editing with technical and editorial input from NB. All authors contributed to the article and approved the submitted version.

## Conflict of Interest

The authors declare that the research was conducted in the absence of any commercial or financial relationships that could be construed as a potential conflict of interest.
