# Supplementary material for: Methods for Detecting Mycobacterial Mixed Strain Infections–A Systematic Review
Source: Front Genet. 2020 Dec 21;11:600692. doi: 10.3389/fgene.2020.600692 (PMC7779811; doi:10.3389/fgene.2020.600692)
Supplement: Supplementary file 1 [file Table_1.pdf]

Supplementary Material

Supplementary Table S1: Keywords, index terms, and strings used to search databases for reports on mycobacterial MSIs. Details regarding term selection are described in the materials and methods section of the main manuscript.

Ovid MEDLINE(R) and Epub Ahead of Print, In-Process & Other Non-Indexed Citations, Daily and Versions(R) 1946 to June 9, 2020: Searched June 11, 2020

#	Search string	Results
1	exp Whole Genome Sequencing/	7158
2	exp Bacteriophage Typing/	4116
3	((Bacteriophage or phage) adj3 typing).ab,ti,kf.	2182
4	IS6110 RFLP.ab,ti,kf.	261
5	MIRU-VNTR.ab,ti,kf.	649
6	Mycobacterial interspersed repetitive unit-variable number tandem repeat typing.ab,ti,kf.	31
7	VNTR.ab,ti,kf.	4993
8	varia* number* tandem* rep* typ*.ab,ti,kf.	91
9	MIRU.ab,ti,kf.	817
10	Mycobacter* interspers* rep* unit*.ab,ti,kf.	614
11	(molecular adj2 (detect* or typing or method* or technique* or diagnos*)).ab,ti,kf.	72323
12	whole genom* sequenc*.ab,ti,kf.	18071
13	(assay adj2 (genom* or molecul*)).ab,ti,kf.	3169
14	typing.ab,ti.	51409
15	1 OR 2 OR 3 OR 4 OR 5 OR 6 OR 7 OR 8 OR 9 OR 10 OR 11 OR 12 OR 13 OR 14	149327
16	exp Mycobacterium/	91603
17	exp Mycobacterium Infections, Nontuberculous/	34845
18	exp Tuberculosis/	191333
19	Coinfection/	10498
20	"Mycobacteri* tuberculosis".ab,ti.	48894
21	Mycobacter*.ab,ti,kf.	97999
22	"m. tuberculosis".ab,ti.	18616
23	"m. africanum".ab,ti.	267
24	"m. bovis".ab,ti.	4581
25	"mycobacteri* bovis".ab,ti.	7148
26	"m. caprae".ab,ti.	98
27	"m. orygis".ab,ti.	11
28	"m. simiae".ab,ti.	211
29	"M. abscessus".ab,ti.	1122
30	"M. chelonae".ab,ti.	616
31	"M. fortuitum".ab,ti.	1132
32	"mycobacteri* gordonae".ab,ti.	320
33	"M. intracellulare".ab,ti.	985
34	"m. avium".ab,ti.	3710
35	"mycobacteri* avium".ab,ti.	9189
36	"m. leprae".ab,ti.	3013
37	"mycobacteri* leprae".ab,ti.	4383
38	Nontuberculous.ab,ti.	4118
39	"heteroresistan*".ab,ti.	580
40	coinfection.ab,ti.	9484
41	co-infection.ab,ti.	10853
42	((mixed or multiple or double or simultaneous or different) adj2 (infection* or strain*)).ab,ti.	50700
43	16 OR 17 OR 18 OR 19 OR 20 OR 21 OR 22 OR 23 OR 24 OR 25 OR 26 OR 27 OR 28 OR 29 OR 30 OR 31 OR 32 OR 33 OR 34 OR 35 OR 36 OR 37 OR 38 OR 39 OR 40 OR 41 OR 42	356175
44	15 AND 43	8932

Supplementary Table S1 (continued)

Ovid MEDLINE(R) and Epub Ahead of Print, In-Process & Other Non-Indexed Citations, Daily and Versions(R) 1946 to August 24, 2020: Revised search to polyclonal and only retrieve results including this new term: Searched August 25, 2020

#	Search string	Results
1	exp Whole Genome Sequencing/	8018
2	exp Bacteriophage Typing/	4117
3	((Bacteriophage or phage) adj3 typing).ab,ti,kf.	2187
4	IS6110 RFLP.ab,ti,kf.	260
5	MIRU-VNTR.ab,ti,kf.	654
6	Mycobacterial interspersed repetitive unit-variable number tandem repeat typing.ab,ti,kf.	31
7	VNTR.ab,ti,kf.	5024
8	varia* number* tandem* rep* typ*.ab,ti,kf.	91
9	MIRU.ab,ti,kf.	822
10	Mycobacter* interspers* rep* unit*.ab,ti,kf.	616
11	(molecular adj2 (detect* or typing or method* or technique* or diagnos*)).ab,ti,kf.	73468
12	whole genom* sequenc*.ab,ti,kf.	18763
13	(assay adj2 (genom* or molecul*)).ab,ti,kf.	3221
14	typing.ab,ti.	50406
15	polyclonal:ti,ab,kw	52613
16	1 OR 2 OR 3 OR 4 OR 5 OR 6 OR 7 OR 8 OR 9 OR 10 OR 11 OR 12 OR 13 OR 14 OR 15	200497
17	exp Mycobacterium/	92185
18	exp Mycobacterium Infections, Nontuberculous/	34993
19	exp Tuberculosis/	192193
20	Coinfection/	10824
21	"Mycobacteri* tuberculosis".ab,ti.	45244
22	Mycobacter*.ab,ti,kf.	98848
23	"m. tuberculosis".ab,ti.	18600
24	"m. africanum".ab,ti.	267
25	"m. bovis".ab,ti.	4617
26	"mycobacteri* bovis".ab,ti.	6847
27	"m. caprae".ab,ti.	101
28	"m. orygis".ab,ti.	12
29	"m. simiae".ab,ti.	213
30	"M. abscessus".ab,ti.	1131
31	"M. chelonae".ab,ti.	619
32	"M. fortuitum".ab,ti.	1138
33	"mycobacteri* gordonae".ab,ti.	322
34	"M. intracellulare".ab,ti.	984
35	"m. avium".ab,ti.	3710
36	"mycobacteri* avium".ab,ti.	9163
37	"m. leprae".ab,ti.	3008
38	"mycobacteri* leprae".ab,ti.	4270
39	Nontuberculous.ab,ti.	3970
40	"heteroresistan*".ab,ti.	574
41	coinfection.ab,ti.	9286
42	co-infection.ab,ti.	10635
43	((mixed or multiple or double or simultaneous or different) adj2 (infection* or strain*)).ab,ti.	50700
44	17 OR 18 OR 19 OR 20 OR 21 OR 22 OR 23 OR 24 OR 25 OR 26 OR 27 OR 28 OR 29 OR 30 OR 31 OR 32 OR 33 OR 34 OR 35 OR 36 OR 37 OR 38 OR 39 OR 40 OR 41 OR 42 OR 43	358223
45	16 AND 44	9825
46	1 or 2 or 3 or 4 or 5 or 6 or 7 or 8 or 9 or 10 or 11 or 12 or 13 or 14	148522
47	44 AND 46	8930
48	45 NOT 47	895

Supplementary Table S1 (continued)

EMBASE (Elsevier): Searched June 11, 2020

#	Search string	Results
1	'mycobacterium'/exp	125,262
2	'mycobacteriosis'/exp	310,109
3	'tuberculosis'/exp	264,147
4	'mixed infection'/exp	35,894
5	'mycobacteri* tuberculosis':ti,ab,kw	54,301
6	mycobacter*:ti,ab,kw	113,620
7	'm. tuberculosis':ti,ab,kw	22,305
8	'm. bovis':ti,ab,kw	4,882
9	'm. caprae':ti,ab,kw	89
10	'm. leprae':ti,ab,kw	3,742
11	nontuberculous:ti,ab,kw	6,133
12	heteroresistan*:ti,ab,kw	703
13	'mixed infection':ti,ab,kw	4,176
14	'm. canetti':ti,ab,kw	13
15	'm. ulcerans':ti,ab,kw	648
16	#1 OR #2 OR #3 OR #4 OR #5 OR #6 OR #7 OR #8 OR #9 OR #10 OR #11 OR #12 OR #13 OR #14 OR #15	388,584
17	'whole genome sequencing'/exp	17,608
18	'bacteriophage typing'/exp	3,993
19	'mycobacterial interspersed repetitive unit-variable number tandem repeat typing':ti,ab,kw	28
20	'miru vntr':ti,ab,kw	771
21	vntr:ti,ab,kw	6,275
22	'varia* number* tandem* rep* typ*':ti,ab,kw	92
23	'mycobacter* interspers* rep* unit*':ti,ab,kw	656
24	'whole genom* sequenc*':ti,ab,kw	22,508
25	(molecular NEAR/2 (detect* OR typing OR method* OR technique* OR diagnos*)):ti,ab,kw	96,957
26	(assay NEAR/2 (genom* OR molecul*)):ti,ab,kw	4,779
27	((bacteriophage OR phage) NEAR/3 typing):ti,ab,kw	2,159
28	typing:ti,ab,kw	68,129
29	miru:ti,ab,kw	983
30	#17 OR #18 OR #19 OR #20 OR #21 OR #22 OR #23 OR #24 OR #25 OR #26 OR #27 OR #28 OR #29	194,819
31	#16 AND #30	7,501

Supplementary Table S1 (continued)

EMBASE (Elsevier): Revised search to polyclonal and only retrieve results including this new term: Searched August 25, 2020

#	Search string	Results
1	'mycobacterium'/exp	126,409
2	'mycobacteriosis'/exp	312,545
3	'tuberculosis'/exp	266,260
4	'mixed infection'/exp	36,795
5	'mycobacteri* tuberculosis':ti,ab,kw	54,980
6	mycobacter*:ti,ab,kw	114,906
7	'm. tuberculosis':ti,ab,kw	22,634
8	'm. bovis':ti,ab,kw	4,985
9	'm. caprae':ti,ab,kw	97
10	'm. leprae':ti,ab,kw	3,761
11	nontuberculous:ti,ab,kw	9,244
12	heteroresistan*:ti,ab,kw	762
13	'mixed infection':ti,ab,kw	4,214
14	'm. canetti':ti,ab,kw	13
15	'm. ulcerans':ti,ab,kw	655
16	#1 OR #2 OR #3 OR #4 OR #5 OR #6 OR #7 OR #8 OR #9 OR #10 OR #11 OR #12 OR #13 OR #14 OR #15	422,591
17	'whole genome sequencing'/exp	18,909
18	'bacteriophage typing'/exp	3,998
19	'mycobacterial interspersed repetitive unit-variable number tandem repeat typing':ti,ab,kw	30
20	'miru vntr':ti,ab,kw	781
21	vntr:ti,ab,kw	6,340
22	'varia* number* tandem* rep* typ*':ti,ab,kw	94
23	'mycobacter* interspers* rep* unit*':ti,ab,kw	661
24	'whole genom* sequenc*':ti,ab,kw	23,411
25	(molecular NEAR/2 (detect* OR typing OR method* OR technique* OR diagnos*)):ti,ab,kw	98,652
26	(assay NEAR/2 (genom* OR molecul*)):ti,ab,kw	4,866
27	((bacteriophage OR phage) NEAR/3 typing):ti,ab,kw	2,161
28	typing:ti,ab,kw	68,784
29	miru:ti,ab,kw	993
30	polyclonal:ti,ab,kw	62,078
31	#17 OR #18 OR #19 OR #20 OR #21 OR #22 OR #23 OR #24 OR #25 OR #26 OR #27 OR #28 OR #29 OR #30	259,744
32	#16 AND #31	9628
33	#17 OR #18 OR #19 OR #20 OR #21 OR #22 OR #23 OR #24 OR #25 OR #26 OR #27 OR #28 OR #29	195,592
34	#16 AND #33	8917
35	#32 NOT #34	711
